# An Oxalyl-CoA Dependent Pathway of Oxalate Catabolism Plays a Role in Regulating Calcium Oxalate Crystal Accumulation and Defending against Oxalate-Secreting Phytopathogens in *Medicago truncatula*

**DOI:** 10.1371/journal.pone.0149850

**Published:** 2016-02-22

**Authors:** Justin Foster, Bin Luo, Paul A. Nakata

**Affiliations:** USDA/ARS Children’s Nutrition Research Center, Department of Pediatrics, Baylor College of Medicine, Houston, Texas, 77030–2600, United States of America; Agriculture and Agri-Food Canada, CANADA

## Abstract

Considering the widespread occurrence of oxalate in nature and its broad impact on a host of organisms, it is surprising that so little is known about the turnover of this important acid. In plants, oxalate oxidase is the most well studied enzyme capable of degrading oxalate, but not all plants possess this activity. Recently, an Acyl Activating Enzyme 3 (AAE3), encoding an oxalyl-CoA synthetase, was identified in Arabidopsis. AAE3 has been proposed to catalyze the first step in an alternative pathway of oxalate degradation. Whether this enzyme and proposed pathway is important to other plants is unknown. Here, we identify the *Medicago truncatula* AAE3 (*Mt*AAE3) and show that it encodes an oxalyl-CoA synthetase activity exhibiting high activity against oxalate with a Km = 81 ± 9 μM and Vmax = 19 ± 0.9 μmoles min^-1^mg protein^-1^. GFP-*Mt*AAE3 localization suggested that this enzyme functions within the cytosol of the cell. *Mtaae3* knock-down line showed a reduction in its ability to degrade oxalate into CO_2_. This reduction in the capacity to degrade oxalate resulted in the accumulation of druse crystals of calcium oxalate in the *Mtaae3* knock-down line and an increased susceptibility to oxalate-secreting phytopathogens such as *Sclerotinia sclerotiorum*. Taken together, these results suggest that AAE3 dependent turnover of oxalate is important to different plants and functions in the regulation of tissue calcium oxalate crystal accumulation and in defense against oxalate-secreting phytopathogens.

## Introduction

Oxalate is an organic acid that plays a pivotal role in a number of biological processes. In plants, oxalate functions in metal tolerance, ion balance, and defense against insects [1–7]. Although the ability to biosynthesize and accumulate oxalate can provide many benefits, uncontrolled or prolonged exposure to this strong organic acid can result in multiple physiological problems which include disruption of membrane integrity, disruption of mitochondrial metabolism, metal precipitation, and free radical formation [8].

Some pathogens utilize the toxic attributes of oxalate in their attack or invasion of hosts. For example, the phytopathogen *Sclerotinia sclerotiorum* secretes oxalate, a known virulence factor, to assist in the successful colonization of the plant tissue. The secreted oxalate is thought to aid in colonization by stimulating stomatal opening, interfering with cell wall structure, inducing low pH activated pectolytic enzymes, chelating cations, and acting as an elicitor of programmed cell death [9–17].

Given the deleterious activity of this acid, plants must carefully regulate tissue oxalate levels in order to maintain proper cellular metabolism and overall plant health. Some plants have been shown to contain an enzyme, oxalate oxidase, which breaks down oxalate to CO_2_ and H_2_O_2_ [18, 19]. This enzyme is part of the germin protein family. Localization studies have shown that oxalate oxidase is targeted to the cell wall and plays a role in stress responses such as defending plants against oxalate secreting fungal phytopathogens (e.g., *S*. *sclerotiorum*). Oxalate oxidase activity has been detected in monocots [20, 21] such as wheat and barley, but in dicots, this activity appears absent [22, 23] suggesting the possible existence of an alternative mechanism to degrade oxalate.

An alternative pathway of oxalate degradation was proposed in plants back in 1961 by Giovanelli and Tobin [24]. Using radiolabeled ^14^C-oxalate and partially purified enzyme extracts from pea (*Pisum sativum*) seeds, Giovanelli and Tobin proposed a pathway in which oxalate was degraded to CO_2_ in a CoA and ATP-dependent manner. With no genes identified to support the existence of this pathway of oxalate catabolism the pathway has remained overlooked for over 50 years. Recently, this has changed with the discovery that the Arabidopsis AAE3 gene encoded the elusive oxalyl-CoA synthetase that is capable of catalyzing the first step in the CoA-dependent pathway of oxalate catabolism [25]. AAE3 is a member of a large superfamily of AAEs in Arabidopsis [26, 27]. Such a catabolic pathway in Arabidopsis is significant since it does not possess oxalate oxidase activity nor does it actively accumulate an appreciable amount of oxalate. In contrast, other plants such as *M*. *truncatula* readily accumulate large amounts of oxalate which is found in the form of the calcium oxalate crystal where it helps defend the plant against chewing insects [28, 29]. It is unknown whether such calcium oxalate accumulating plants also have a need to utilize the CoA-dependent pathway of oxalate catabolism or whether this pathway is exclusive to Arabidopsis, a non-crystal accumulating plant.

In this study we investigate whether AAE3-dependent oxalate turnover occurs in *M*. *truncatula* and assess its biological function in this calcium oxalate crystal forming plant. Bioinformatic, biochemical, genetic, and molecular studies revealed that *Mt*AAE3 encodes an oxalyl-CoA synthetase capable of catalyzing the conversion of oxalate to oxalyl-CoA. Radiolabeled oxalate tracer studies suggest that the *Mt*AAE3 catalyzes the first step in the CoA and ATP-dependent pathway of oxalate degradation. Fungal growth and calcium oxalate assays support biological roles for MtAAE3 in defense against oxalate-secreting phytopathogens and in the regulation of calcium oxalate crystal accumulation.

## Materials and Methods

### Plant material and growth conditions

Seeds of *M*. *truncatula* ecotype R108 were removed from their pods, nicked with a razorblade, and germinated on agar plates. Germinated plants were grown in MetroMix 300 soil mix (SunGro Horticulture, Agawam, MA) under controlled greenhouse conditions at 24°C. Natural light was supplemented with artificial lighting using a 16 hour day/8 hour night photoperiod. *Arabidopsis thaliana* ecotype Columbia seeds were sterilized by soaking in 30% bleach with 0.1% Triton X-100, rinsed five times with sterile water and plated on Murashige and Skoog (MS) medium, pH 5.7 [30], supplemented with 1% sucrose and 0.8% agar. Germinated plants were grown in Sunshine professional growing mix (SunGro Horticulture, Agawam, MA) in environmentally controlled growth chambers at 22°C with a 16 hour day/8 hour night photoperiod. In the MtAAE3 oxalate induction studies, the germinated *M*. *truncatula* plants were grown hydroponically as previously described [31]. The seedlings then were transferred to water (control) or oxalate (1 mM) and roots and shoots harvested and frozen in liquid nitrogen until use. In the seed germination study, seeds of wild type (WT), *Ataae3*, and *Ataae3/MtAAE3* were sterilized and planted as described above. The germination rate was determined after one week.

### *MtAAE3* cDNA isolation

Total RNA was extracted from leaves of 4-week old *M*. *truncatula* plants using TRIzol reagent (Life Technologies, Thermo Fisher Scientific, Grand Island, NY) according to the manufacturer’s instructions. Total RNA was used for first-stand cDNA synthesis using oligo (dT) and Superscript III first strand synthesis supermix (Thermo Fisher Scientific). The *MtAAE3* coding sequence was amplified by PCR using a 4 μl aliquot of the reverse transcription reaction, gene specific primers 5’-ATGGAAACCGCTACAACCCTCAC-3’ and 5’-TGAAGCTTGAGAGACAAAGTGTTC -3’, and Platinum Taq DNA polymerase, High Fidelity (Thermo Fisher Scientific) according to manufacturer’s instructions. All hybridization steps were performed using a PTC-200 thermal cycler (Bio-Rad, Hercules, CA). The PCR product was cloned using the pGEM-T Easy kit (Promega, Madison, WI, USA) according to manufacturer’s instructions and verified by DNA sequencing (Lonestar Labs, Houston, TX, USA).

### His-tagged *Mt*AAE3 recombinant protein purification

To create a His-tagged *Mt*AAE3 fusion protein, the full-length *Mt*AAE3 cDNA was amplified by PCR using the primers, 5-CATATGCACCACCACCACCACCACAGCCAGGAAACCGCTACAACCCTCAC-3 which introduced a *Nde*I site and six histidine residues on its N-terminus and 5-CGAGCTCTCAAGGCTTGAGAGACAAAGTGTT-3 which contained an end terminal *Sac*I site. The PCR product was ligated into the plasmid vector pGEM-T Easy (Promega) and sequenced. The *Nde*I/*Sac*I His-*Mt*AAE3 fragment was transferred from the pGEM-T Easy vector into the protein expression vector Pet-29a (Novagen, EMD Millipore, Billerica, MA USA) using the same restriction sites. *Escherichia coli* strain BLR (DE3) competent cells (Novagen) were transformed with the N-terminal His-tagged *Mt*AAE3 expression vector. A small culture was grown overnight at 37°C and used to inoculate 500 mL of Luria-Bertani medium. The culture was incubated at 37°C until it reached an OD_600nm_ of 0.4. To induce expression, IPTG was added to 1 mM, and the culture was grown for an additional 4 hours at 30°C. The cells were then collected by centrifugation and the cell pellet frozen.

Affinity purification of the His-tagged *Mt*AAE3 was performed as described in the Qiagen protein purification kit manual (Valencia, CA, USA). In brief, the bacterial cell pellet was thawed for 15 min on ice. The thawed cells then were resuspended in lysis buffer (50 mM NaH_2_PO_4_, 300 mM NaCl, and 10 mM imidazole, pH 8.0) supplemented with lysozyme (1 mg/mL) and benzonase and incubated on ice for an additional 30 min followed by sonication to lyse the cells. The extract then was cleared by centrifugation at 10,000 x g for 25 min at 4°C. The supernatant was collected and loaded onto a column packed with nickel-nitriloacetic acid agarose to bind the His-tagged *Mt*AAE3. The column was washed with wash buffer (50 mM NaH_2_PO_4_, 300 mM NaCl, and 20 mM imidazole at pH 8) and eluted using 1 mL of elution buffer (50 mM NaH_2_PO_4_, 300 mM NaCl, and 250 mM imidazole, pH 8.0). Salts were removed by passing the protein sample through a column packed with Sephadex G-25 (Sigma-Aldrich, St. Lous, MO) and equilibrated with 100 mM Tris-HCl, pH 7.5. The protein concentration of the eluate, approximately 1mL at five μg/μL, was determined by Bradford assay [32]. An estimation of the molecular weight and purity of the affinity purified *Mt*AAE3 sample was assessed by SDS-polyacrylamide gel and Coomassie Brilliant Blue R 250 staining (Bio-Rad).

### *Mt*AAE3 enzyme activity and kinetics

*Mt*AAE3 enzyme activity was determined by a coupled enzyme assay as previously described [25]. Briefly, the assay was initiated by the addition of 5 μg of the purified *Mt*AAE3 to the buffered reaction mixture (0.1 M Tris-HCl, pH 8.0, or 0.1 M NaPO_4_, pH 8.0, 2 mM DTT, 5 mM ATP, 10 mM MgCl_2_, 0.5 mM CoA, 0.4 mM NADH, 1 mM phosphoenol-pyruvate, and 10 units each of myokinase, pyruvate kinase, and lactate dehydrogenase and the carboxylic acid substrate) in a final volume of 800 μL. The reaction was monitored by measuring the oxidation of NADH at 340 nm using a Varian Cary 50 spectrophotometer (Agilent Technologies, Santa Clara, CA, USA). A range of oxalate concentrations were employed to determine K_m_ and V_max_ while the optimal pH and substrate specificity was determined using 300 μM of oxalate.

### *Mtaae3* RNAi knock-down line

To create the RNAi knock-down construct a hairpin loop, containing two complementary *MtAAE3* sequences separated by Restricted Tobacco etch virus Movement (RTM) intron (27), was constructed. The RTM was amplified from Arabidopsis using the primers, 5- CCCTCTAGAACGTTGTAAGTCTGATTTTTGAC-3 and 5- CCCGCGGCCGCTCTATCTGCTGGGTCCAAATC-3, which introduced *Xba*I and *Not*I on the 5’ and 3’ end of the amplified fragment, respectively. The resulting fragment was inserted into pBluescript II KS (+) vector (Agilent Technologies) via *Not*I and *Xba*I sites to make the pINTRON vector. A 225 bp segment of the *MtAAE3* gene was amplified using the *MtAAE3* cDNA as template and the primers, 5-TGAGCTCATTTCCATTCATATTTATCACCA-3 and 5-TGCGGCCGCGACATCGTTGGGTTTGATTCCA-3 which introduce a *Sac*I and *Not*I site on the 5’and 3’ end of the amplified fragment, respectively. The reverse complement of this 225 bp segment was generated using the same template cDNA and the primers, 5-TTCTAGAGACATCGTTGGGTTTGATTCCA-3 and 5-TGGATCCATTTCCATTCATATTTATCACCA-3 which introduce a *Xba*I and *Bam*HI site on the 5’ and 3’ end of the fragment, respectively. The *Sac*I-*Not*I *MtAAE3* fragment was cloned into the pIntron vector after digesting with same restriction sites. After amplification of this construct in DH5α the *Bam*HI-*Xba*I *Mt*AAE3 fragment was cloned into the corresponding sites of the vector. In this construct the two complementary *MtAAE3* sequences were separated by 143 bp of Restricted Tobacco etch virus Movement (RTM) intron to create a hairpin loop [33]. The *MtAAE3*-intron-*MtAAE3* sequence was liberated by digestion with *Bam*HI/*Sac*I and used to replace the GUS gene in the p3300/p*Mt*AAE3::GUS expression construct created using pCAMBIA vector (see below) [34]. The resulting RNAi expression construct was transformed into *A*. *tumefaciens* strain EHA105 by electroporation and transformed into WT *M*. *truncatula* R108 [34].

### *M*. *truncatula* transformation

*M*. *truncatula* R108 [35] plants were transformed with *A*. *tumefaciens* strain EHA105 harboring the various binary constructs using an embryogenesis protocol as previously reported [36]. The T1 plants were grown in the greenhouse and the seeds collected. The T2 seeds were germinated and plants grown as described above. Transgenic T2 plants were further selected by spraying with Basta (Finale, AgrEvo Environmental Health, Montvale, NJ, USA).

### Radiolabeled oxalate feeding

Leaf discs of *M*. *truncatula* wild type and RNAi knock-down lines were isolated from 4 week old plants using an 8.5 mm borer. The leaf discs were then placed in an Erlenmeyer flask containing 5 ml of MS media, pH 5.7 [30] supplemented with 0.5% sucrose, 0.05% MES, 500 μM oxalate and 5 μCi of [^14^C]-oxalate (American Radiolabeled Chemicals, St. Louis, MO, USA). A glass vial containing 500 μl of 1M KOH was utilized as a CO_2_ trap and the flask sealed with a neoprene stopper. The flasks were slowly shaken at room temperature for 5 hours and the reaction stopped by the addition of 1 mL of 0.25 M HCl that was injected through the stopper. The leaf disks were shaken for an additional 10 min and the radiolabeled CO_2_ trapped in within the KOH measured using a Tricarb 2500TR liquid scintillation analyzer (Packard Bioscience Co., Meriden, CT).

### Subcellular localization of *Mt*AAE3

A *35S*::*GFP-MtAAE3* construct was generated by first restriction digesting pBI121 (Clontech, Mountain View, CA) with *Hin*dIII and *Sac*I and transferring the 35S::GUS::NOS cassette to pCAMBIA1300 binary vector (CAMBIA, Canberra Australia) using these same sites. This ligation resulted in a p1300/35S::GUS construct. The EGFP gene was modified and amplified by PCR using the primers, 5’-CCCTCTAGAATGGTGAGCAAGGGCGAG-3’ and 5’-CCGGATCCTGGACTTGTACAGCTCGTCCATG-3’, and pEGFP (Clontech, Mountain View, CA, USA) as template. The resulting EGFP fragment then was inserted into p1300/35S::GUS construct via the *Xba* I and *Bam*H I sites generating the construct, p1300/35S::GFP-GUS. The *MtAAE3* coding sequence then was modified and amplified with primers, 5’- TCCCCGGGATGGAAACCGCTACAACCCTCA -3’ and 5’- CGAGCTCTCAAGCTTGAGAGACAAAGTGTT -3’, and used to replace the *GUS* fragment in *p1300/35S*::*GFP* via *Sma*I and *Sac*I sites. This p1300/35S::GFP-*Mt*AAE3 construct was transiently expressed in *N*. *benthamiana* by leaf infiltration [37, 38] with *Agrobacterium tumefaciens*. Protein localization was investigated using a FV300 laser scanning confocal microscope (Olympus America Inc. NY, USA) using an argon laser. A 488 nm excitation and a 505 to 530 nm emission filter set were utilized for GFP observation. Photographs then were arranged into a composite figure using Photoshop software (Adobe Systems Inc., San Jose, CA, USA).

### *MtAAE3* expression analysis

Total RNA was extracted from leaves using TRIzol reagent (Life Technologies) and reverse transcribed with iScript Reverse Transcription Supermix (Bio-Rad). The diluted cDNA was used as template for PCR. *MtAAE3* expression levels were measured using the primers 5’- CTGTCTTGGGCAAAGAATCAG -3’ and 5’- CGGTGAAGAGATACATTGTGC -3’. The *M*. *truncatula Ubiquitin* gene (AC137828_19.4) was used as a reference gene to normalize the cycle threshold value [39]. The ubiquitin gene expression levels were monitored using the primers 5’- GCAGATAGACACGCTGGGA -3’ and 5’- AACTCTTGGGCAGGCAATAA -3’ [39]. Quantitative real time PCR was performed using a Bio-Rad CFX-96 real-time PCR detection system and SYBR Green master mix (Clontech Laboratories) or SYBR Green Supermix (Bio-Rad Laboratories, Hercules, CA, USA) according to the manual protocols. The results were analyzed using CFX manager software. To verify that unique PCR products were amplified, the PCR product was cloned using the pGEM-T Easy kit (Promega, Madison, WI, USA) according to manufacturer’s instructions and verified by DNA sequencing (Lonestar Labs, Houston, TX, USA). To determine specificity a melt curve was done that showed only one peak indicating a specific primer pair.

### Complementation of *Ataae3*

The *35S* promoter in p1300/35S::GFP-*Mt*AAE3 was replaced by the Arabidopsis *AAE3* promoter [25] via *Xba*I and *Bam*HI sites generating the p1300/p*AtAAE3*::GFP-*Mt*AAE3 construct. This construct was utilized for complementing *Ataae3-1* mutant. Arabidopsis transformation was performed using floral-dip method [40]. The transgenic Arabidopsis plants were selected on MS plates containing 50 mg L^-1^ hygromycin.

### Microscopic analysis of calcium oxalate crystal phenotype

Leaf and root samples were harvested from 3–4 week old plants and cleared in 95% (v/v) ethanol. The tissue samples then were equilibrated with water and visually inspected for calcium oxalate crystal deposition using light microscopy and crossed polarizers. Images of whole-tissue mounts were captured using a CCD72 camera mounted on a Zeiss Axiophot light microscope (Carl Zeiss Microscopy, Jena, Germany).

### Measurement of oxalate content in the plant

Oxalate concentrations were measured by HPLC. Leaves and roots were harvested from three independently grown sets of plants and ground in liquid nitrogen. Oxalate extraction was performed as described previously [31] and the samples filtered (0.2 μm) and analyzed for oxalate by HPLC (Agilent 1100) coupled to a photodiode array detector (Agilent 1100) at 210nm with a Bio-Rad Aminex HPX-87H ion exclusion column (300 X 7.8 mm), eluted with 5 mM H_2_SO_4_ with a flow rate of 0.6 mL/min at 35°C [25]. External standards of oxalate were used to determine sample oxalate concentrations.

### Fungal growth assays

*S*. *sclerotiorum* was grown on potato dextrose agar (PhytoTechnology Laboratories, Shawnee Mission, KS), and a 2 cm^2^ region of hyphae was cut and transferred to 5 mL liquid culture of 0.5% potato dextrose broth and incubated for 2 d at room temperature with shaking. The 5 mL culture was then diluted 1:10 with fresh 0.5% potato dextrose broth and homogenized using a polytron. Leaves from 7-week old *M*. *truncatula* plants were then cut off the plant by severing the petiole and placed on wet paper. Uniform sized discs of filter paper were created using a hole-punch. Each disc was cut into four equal pie-shaped pieces. Each pie-shaped piece of paper was inoculated with 5 μL of fungal suspension which was then placed on each leaf. After 48 h, leaves were photographed and the lesion areas were measured using the ImageJ software [41].

## Results and Discussion

### *MtAAE3* encodes an oxalyl-CoA synthetase

Although oxalic acid is common in nature and has a broad impact on plants our understanding of the mechanisms regulating its turnover remains incomplete. Recently, a novel pathway of oxalate catabolism was suggested in Arabidopsis [25]. The existence of this catabolic pathway is supported by the discovery of an oxalyl-CoA synthetase encoded by the *A*. *thaliana* AAE3 (*At*AAE3) which has been shown to catalyze the first step in this pathway [25]. As a step toward determining whether this enzyme and proposed pathway of oxalate catabolism is important to other plants a database search was conducted for ORFs encoding amino acid sequences similar to the translated *At*AAE3. This bioinformatics search led to the identification of the Medtr3g035130 ORF which shares 75% amino acid sequence identity with *At*AAE3.

**Fig 1:**
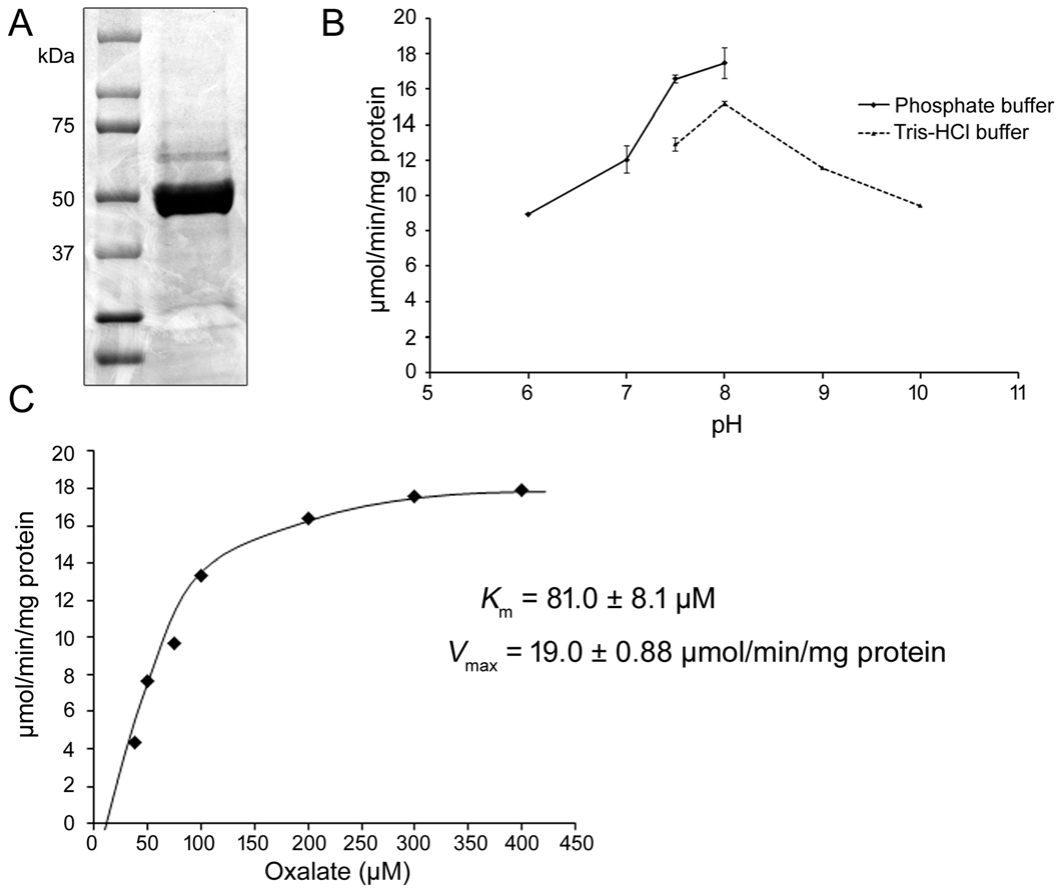
Biochemical Analysis of *Mt*AAE3. (A) SDS-PAGE gel of nickel-affinity purified His-*Mt*AAE3 protein (right) and molecular weight markers (left). (B) Optimum pH for *Mt*AAE3 was performed with 300 μM oxalate using two buffer systems; potassium phosphate pH 6–8 and Tris-HCl pH 7.5–10. (C) Kinetic analysis of *Mt*AAE3 using a range of oxalate concentrations. K_m_ and V_max_ were determined from non-linear regression to the Michaelis-Menten equation for concentrations up to 400 μM oxalate.

To determine if Medtr3g035130 encodes an oxalyl-CoA synthetase, the HIS-tagged-fusion of this protein was constructed, expressed in *E*. *coli*, and purified by nickel-affinity chromatography. The recombinant protein was estimated to be >90% pure based on fractionation profiles generated by SDS-PAGE (Fig 1A). Spectrophotometric coupled-enzyme assay [42] revealed that Medtr3g035130 encoded a *M*. *truncatula* AAE3 (*Mt*AAE3) capable of converting oxalate to oxalyl-CoA. The *Mt*AAE3 protein showed activity against oxalate over a wide pH range with an optimum at a pH of 8.0 (Fig 1B). Previous work in Arabidopsis [43] has shown a cytosolic pH of 7.3. We hypothesize that the cytosolic pH of the cells in *M*. *truncatula* would be similar suggesting that the measured *Mt*AAE3 pH optimum of 8.0 would be physiologically relevant. Interestingly, *Saccharomyces cerevisiae* AAE3 (*Sc*AAE3) has been shown to be associated with the peroxisome by proteomic analysis [44]. The perixosomal lumen has been shown to be alkaline with a pH of 8.2 [45]. At pH 8.0 and with saturating concentrations of CoA and ATP the enzyme displayed Michaelis-Menten kinetics with respect to oxalate concentration up to 400 μM (Fig 1C). Using this data a V_max_ of 19 ±0.9 μmoles min^-1^mg protein^-1^ and a K_m_ of 81.0 ± 8.1 μM was calculated. The V_max_ for *Mt*AAE3 is higher than the 11.4±1.0 μmoles min^-1^mg protein^-1^ reported for *At*AAE3 [25] and the 12.0±1.0 μmoles min^-1^mg protein^-1^reported for the *Sc*AAE3 that was shown to be important for the metabolism of oxalate in yeast [46].

### *Mt*AAE3 is required for the production of CO_2_ from the catabolism of oxalate

**Fig 2:**
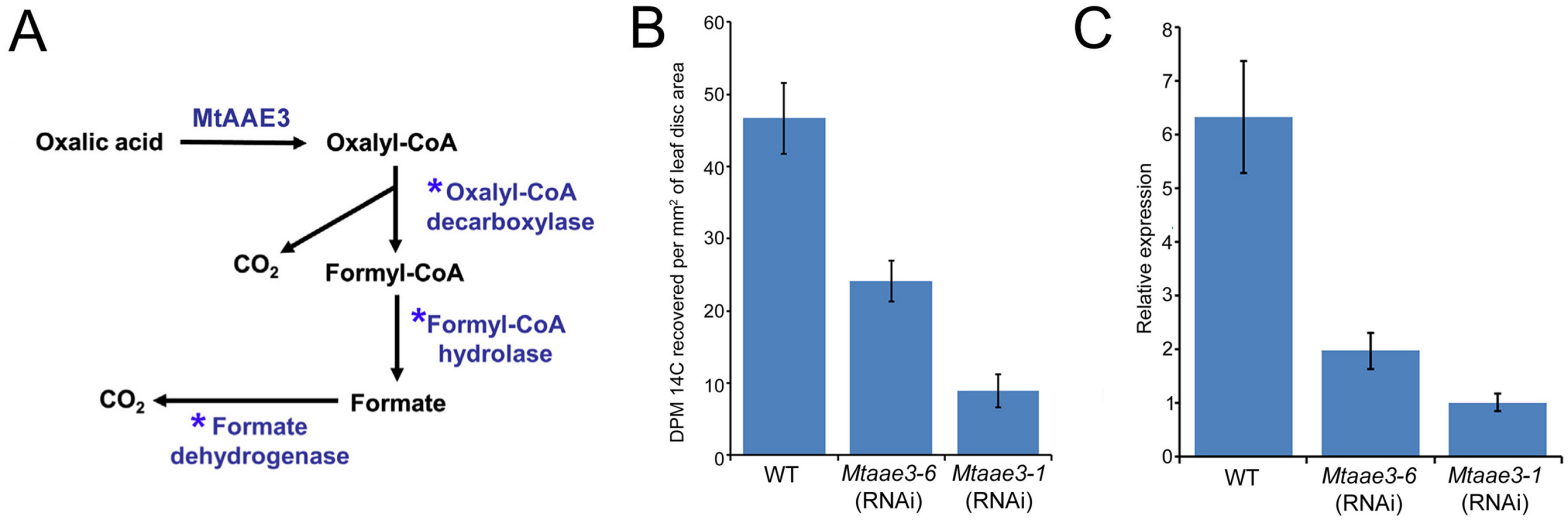
Measurement of oxalate degradation to CO_2_. (A) Schematic of the proposed pathway of oxalate catabolism in *M*. *truncatula*. Asterisks denote enzymatic steps remaining to be validated. (B) Radiolabeled CO_2_ measurements. WT and *Mtaae3* knock-down leaf pieces were fed with 2.5 μCi of [^14^C]-oxalate along with 300 μM non-labeled oxalate. The ^14^CO_2_ evolved was captured using 1M KOH and the relative radioactivity was measured. (C) Relative *MtAAE3* transcript levels in leaves of *Mtaae3* knock-down lines compared to WT as measured by qRT-PCR. Ubiquitin was used as the reference gene.

A novel pathway of oxalate degradation (Fig 2A) that proceeds from oxalate to oxalyl-CoA to formyl-CoA to formate and eventually to CO_2_ has been proposed in plants [25, 47]. The first step in this pathway has been shown in Arabidopsis to be catalyzed by AAE3 [25]. To assess whether *Mt*AAE3 catalyzes the first step in a similar pathway in *M*. *truncatula*, radiolabeled oxalate feeding experiments were conducted using WT and a *Mtaae3* RNAi knock-down line. The RNAi knock-down line was generated by transforming WT *M*. *truncatula* with a construct expressing inverted segments of the *Mt*AAE3 coding region (stem) separated by a short segment of the Tobacco Etch Virus intron (hairpin loop). WT plants were found capable of degrading the ^14^C-oxalate yielding ^14^CO_2_. The *Mtaae3* knock down however, had an approximate 50% reduction in ^14^CO_2_ emissions (Fig 2B). This reduction in ^14^CO_2_ emissions correlated with the reduction in *MtAAE3* gene expression compared to ubiquitin as measured by quantitative RT-PCR (Fig 2C). Overall, these findings support a role for *Mt*AAE3 in catalyzing the first step in a pathway of oxalate degradation to CO_2_ in *M*. *truncatula*. In further support, bioinformatics analysis indicated the presence of a putative *M*. *truncatula* homolog of the Arabidopsis oxalyl-CoA decarboxylase which is the enzyme responsible for catalyzing the second step in this novel pathway in Arabidopsis (Foster et al., unpublished). The putative *M*. *truncatula* homolog shows 79% identity to the Arabidopsis oxalyl-CoA decarboxylase (Foster et al., unpublished). In contrast to the *Ataae3* mutant, the *Mtaae3* knock-down line did not exhibit any readily identifiable mutant growth and development phenotype. The lack of growth and development phenotype may be a result, at least in part, to the use of a knock-down line rather than a knock-out mutant as in the case of Arabidopsis [25]. Whether the complete absence of *MtAAE3* transcript would result in defects in seed germination and/or mucilage as observed in Arabidopsis remains to be determined.

### *Mt*AAE3 is localized to the cytosol in *Nicotiana*, and complements an Arabidopsis *Ataae3* mutant

**Fig 3:**
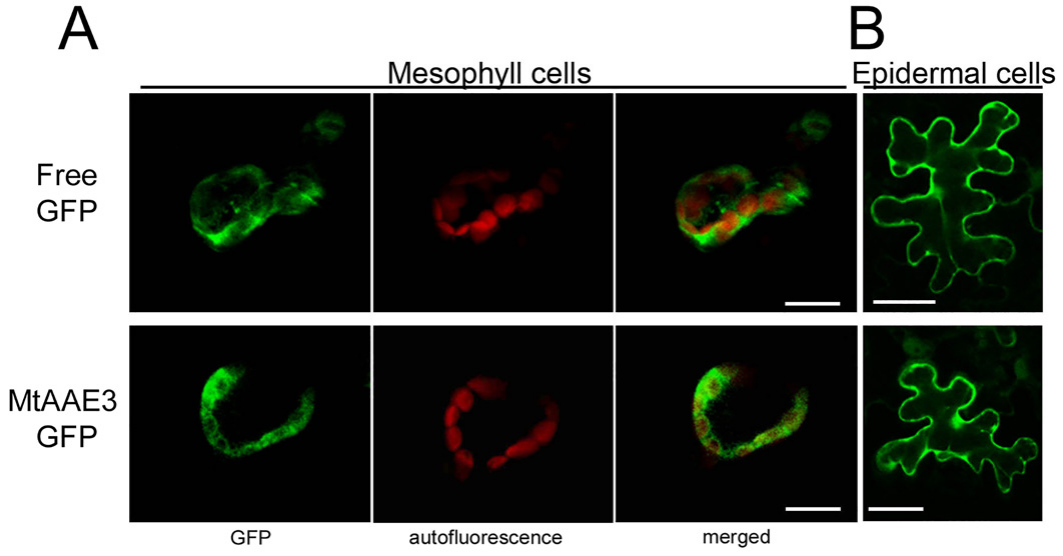
Subcellular localization of *Mt*AAE3. (A) Transient expression of free GFP (top row) and GFP-*Mt*AAE3 (bottom row) in mesophyll leaf cells of *N*. *benthamiana*. GFP fluorescence images (left), chloroplast autofluorescence (middle), and merge of GFP and autofluorescence (right). (B) Transient expression of free GFP (top) and GFP-*Mt*AAE3 (bottom) in *N*. *benthamiana* epidermal leaf cells. Bar = 20 μm

**Fig 4:**
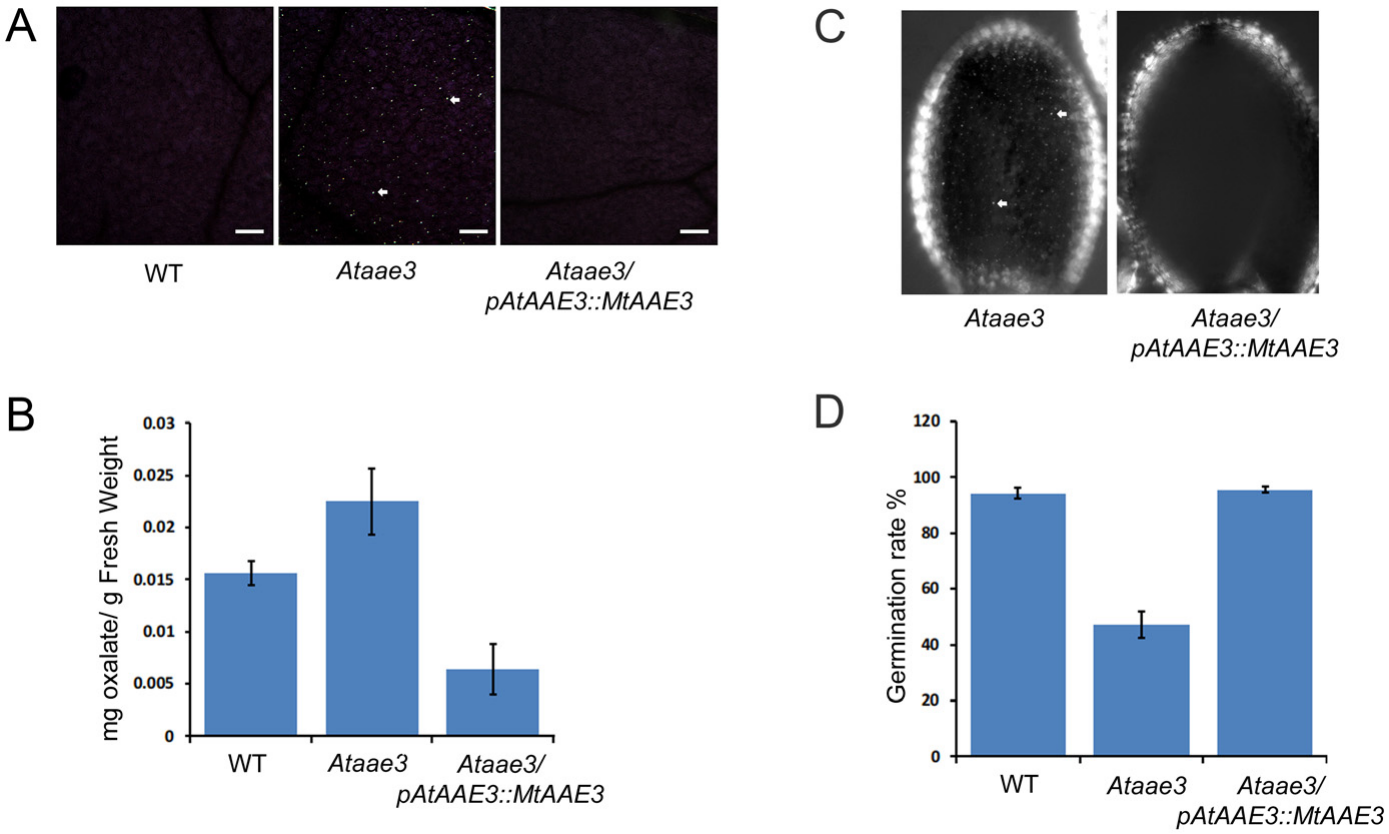
Complementation of *Ataae3* with *Mt*AAE3 under the control of the *AtAAE3* promoter. (A) Calcium oxalate crystal leaf phenotype of WT, *Ataae3*, and *Ataae3*/p*AtAAE3*::GFP-*Mt*AAE3 Arabidopsis. Crystals are bright spots designated by arrows. (B) Oxalate concentrations in leaves from WT, *Ataae3*, and *Ataae3*/p*AtAAE3*::GFP-*Mt*AAE3 Arabidopsis. (C) Complementation of *Ataae3* calcium oxalate crystal seed phenotype through expression of *Mt*AAE3. Crystals are bright spots designated by arrows. (D) Germination of WT, *Ataae3*, and *Ataae3*/p*AtAAE3*::GFP-*Mt*AAE3 seeds. Bar = 50 μm.

To investigate the subcellular localization of MtAAE3, we transiently expressed a GFP-*Mt*AAE3 fusion protein in the leaves of *Nicotiana benthamiana*. Expression of the GFP-*Mt*AAE3 fusion protein was observed by confocal microscopy. As evidenced by its fluorescent pattern in comparison to the pattern exhibited by the free GFP control, the GFP-*Mt*AAE3 fusion protein was observed to reside within the cytoplasm of the cell (Fig 3). Functional validation of this expression construct was achieved in Arabidopsis by GFP-*Mt*AAE3 complementation of *Ataae3* T-DNA mutant (Fig 4). Expression of this GFP-*Mt*AAE3 transgene in *Ataae3* restored the WT phenotype (no crystals) in leaves (Fig 4A) and seeds (Fig 4C). Oxalate measurements confirmed this visual rescue by revealing the expected low tissue oxalate levels in the leaves of the complemented mutants (Fig 4B). Oxalate levels in the complemented mutant were lower than WT presumably due to the higher activity of the *Mt*AAE3 (V_max_ of 19 ±0.9 μmoles min^-1^mg protein^-1^) compared to the *At*AAE3 (V_max_ of 11.4 ±1.0 μmoles min^-1^mg protein^-1^) and possible higher expression of the transgene due to positional effects. The ability to lower tissue oxalate levels raises an interesting point. Oxalate is a known antinutrient in that it binds calcium in a form (calcium oxalate crystal) that renders the calcium unavailable for nutritional absorption by humans and other animals [48, 49]. A reduction in the amount of calcium bound as the oxalate salt has been shown to result in a proportional increase in calcium bioavailability [48, 50, 51]. Thus, the removal of oxalate utilizing AAE3 and its associated pathway of oxalate degradation may be a viable option for the nutritional improvement of plant foods.

Expression of the *Mt*AAE3 in an Arabidopsis *Ataae3* mutant also restored germination rates of the mutant to WT levels (Fig 4D). Poor germination was a characteristic reported for the *Ataae3* mutant [25]. Such a defect was not readily apparent in the *Medicago Mtaae3* knock-down line. Complete knock-out of AAE3 expression may be required before the poor germination phenotype becomes apparent.

### Reduction of *Mt*AAE3 increases susceptibility of *M*. *truncatula* to an oxalate-secreting fungal pathogen

**Fig 5:**
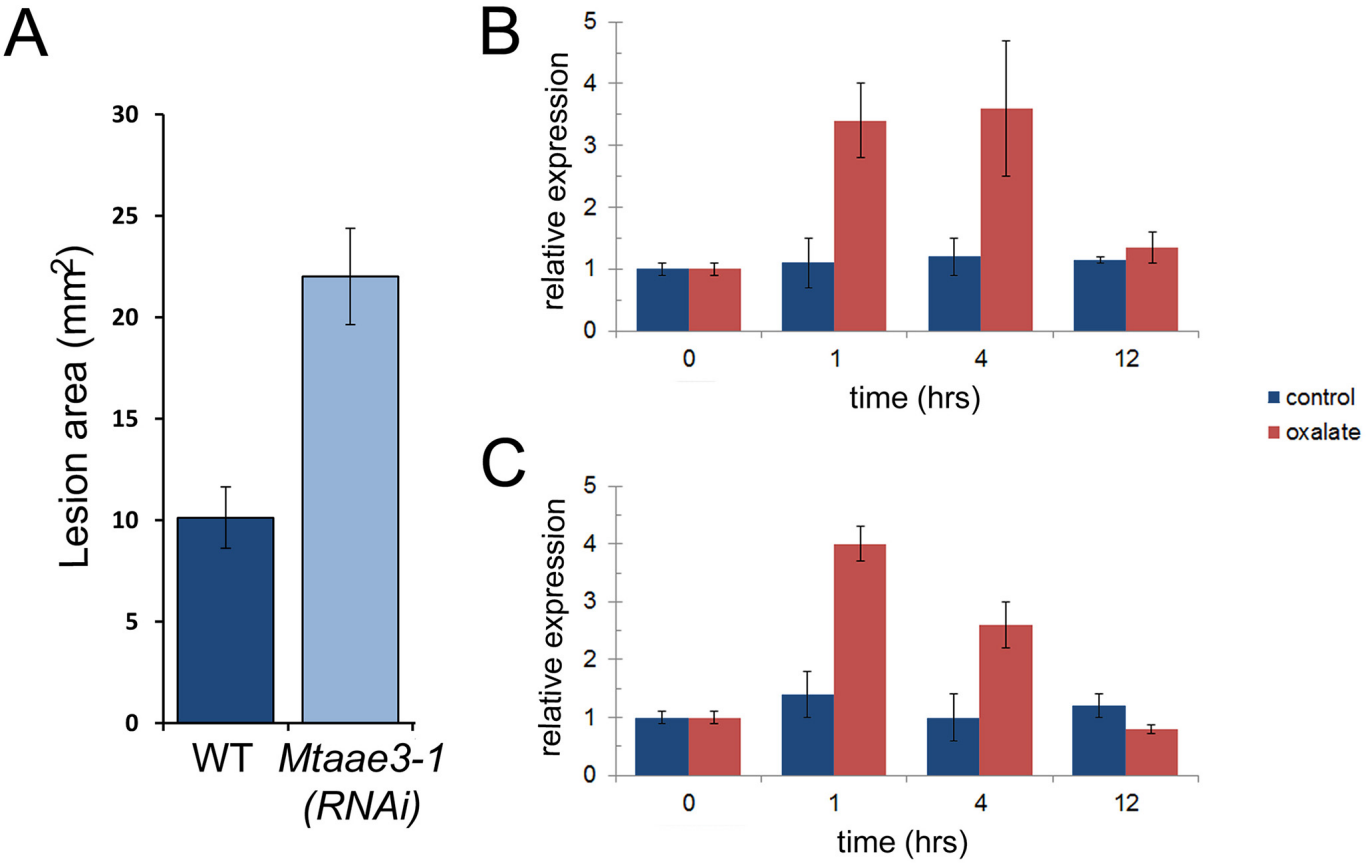
Assessment of *Mt*AAE3 in protection against oxalate-secreting phytopathogens. (A) Measurements of fungal spread in leaves of WT and *Mtaae3-1* knock-down line inoculated with *S*. *sclerotiorum*. Lesion areas were measured 48 h after inoculation. Data are mean ± SE. (B) Quantitative RT-PCR of *MtAAE3* expression in roots from control and oxalate-treated plants over time. (C) Quantitative RT-PCR of *MtAAE3* expression in shoots from control and oxalate-treated plants over time. The cycle threshold values were normalized using the ubiquitin as a reference gene.

Oxalate is a known virulence factor required by certain oxalate-secreting phytopathogens for infection [9, 11, 12, 52]. To investigate a role for *Mt*AAE3 in conferring resistance to oxalate-secreting phytopathogens, leaves of *Medicago* WT and an *Mtaae3* RNAi knock-down line (Fig 5A) were inoculated with *S*. *sclerotiorum*. Lesion areas were measured after a 2 d incubation period. The *Mtaae3-1* knock-down line displayed higher susceptibility to *S*. *sclerotinia* than WT, as indicated by the larger lesion size (Fig 5A). This finding is supported by a similar experiment comparing the susceptibility of the WT Arabidopsis and the *Ataae3* mutant to *S*. *sclerotiorum*. The *Ataae3* was also found to be more susceptible to the oxalate secreting phytopathogen [25].

To determine whether oxalate induced the expression of *MtAAE3*, hydroponically grown WT *M*. *truncatula* was exposed to an exogenous supply of the acid by simply replacing the hydroponic media with 1 mM oxalate. Replacement of the hydroponic solution with water was done as a control. Roots and shoots were then harvested at t = 0, 1, 4, and 12 h time points. RNA was isolated from each tissue set and *MtAAE3* expression assessed by quantitative RT-PCR (Fig 5B and 5C). In each tissue a several-fold increase in *MtAAE3* transcript abundance was detected one hour after oxalate exposure. The *MtAAE3* transcript abundance then declined back to baseline levels by the twelfth hour. Overall, the results support a role for *Mt*AAE3 in reducing the deleterious effects of the phytopathogenic fungus *S*. *sclerotiorum* by catabolizing the virulence factor, oxalate. This benefit underscores the importance of determining which plants utilize this pathway of oxalate inactivation. Such knowledge would be useful in efforts to exploit this pathway in engineering increased resistance to oxalate-secreting phytopathogens. Earlier work supported the potential of this strategy by showing that simple over-expression of AAE3 in an Arabidopsis plant with a functional pathway increased resistance [25]. Whether the over-expression of the subsequent steps in the pathway results in a further enhancement remains to be determined.

### Reduction of *Mt*AAE3 results in the accumulation of druse crystals of calcium oxalate.

**Fig 6:**
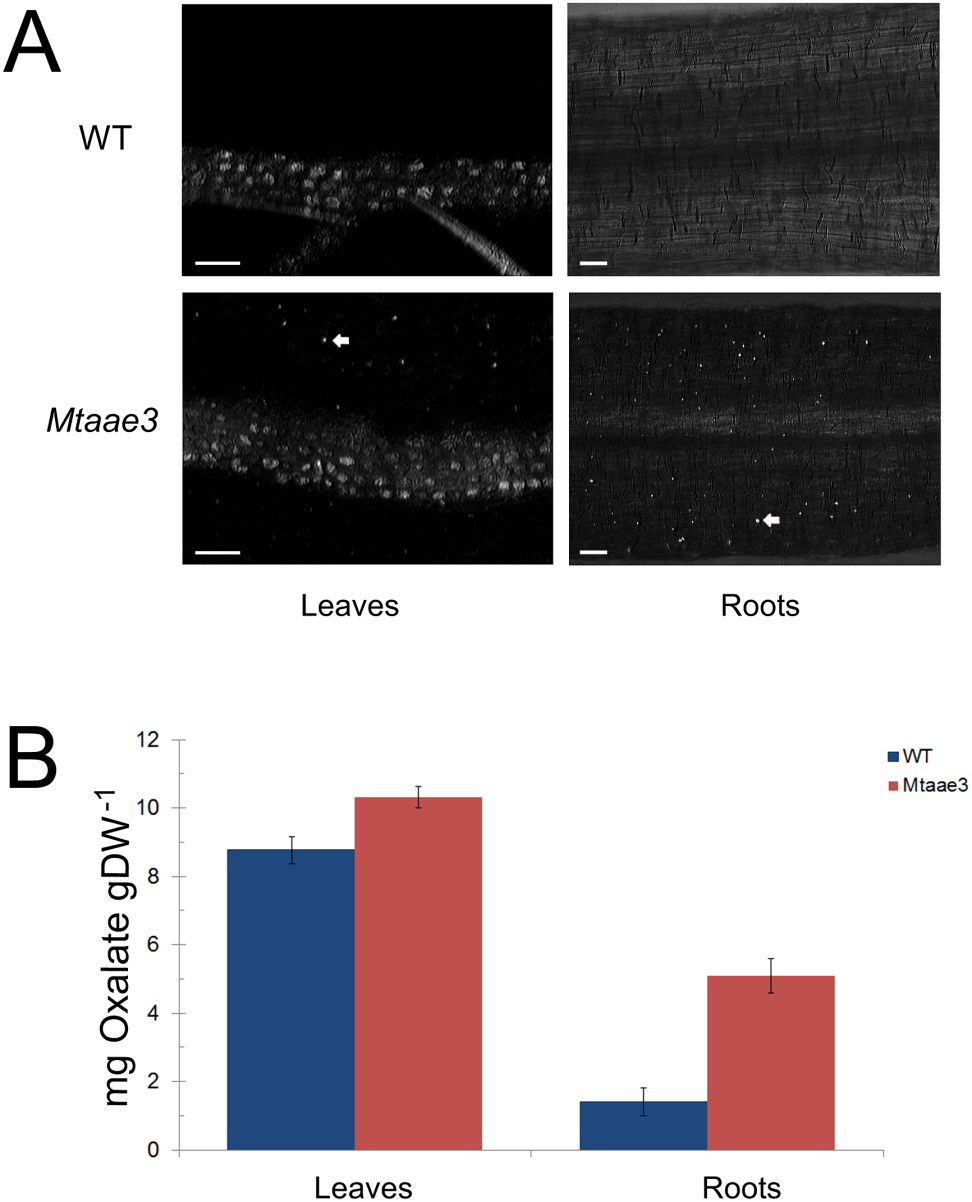
Assessment of *Mt*AAE3 in the regulation of calcium oxalate accumulation. (A) Comparison of the crystal phenotypes in leaf and root from WT and *Mtaae3* knock-down plants. Crystals are bright spots denoted by arrows. (B) Total oxalate in leaf and root from WT and *Mtaae3* RNAi knock-down plants. Bar = 50 μm.

*M*. *truncatula* accumulates prismatic crystals along the vascular strands of secondary veins in leaves, but such crystals are absent in roots (Fig 6A). In correlation with these observations leaves were found to contain oxalate concentrations that were several-fold higher than roots (Fig 6B). To investigate a role for *Mt*AAE3 in regulating calcium oxalate accumulation, oxalate measurements were conducted in leaves and roots from the *Mtaae3-1* knock-down line. Higher oxalate concentrations were measured in both leaves and roots from the *Mtaae3-1* knock-down line compared to the corresponding control tissues (Fig 6B). Oxalate concentrations did not increase; however, to the expected levels if *Mt*AAE3 was regulating prismatic crystal formation. Microscopic examination of these two tissues confirmed that the pattern of prismatic crystal accumulation had not changed in these two tissues. Instead, this examination revealed that the measured increase in oxalate resulted from the accumulation of a second crystal type known as druse within the mesophyll cells of leaves and roots (Fig 6A). Druse crystals have been shown to accumulate in the mesophyll cells of older leaves of *M*. *truncatula* [31]. Microscopic images show that prismatic and druse crystals accumulate in different cell-types (Fig 6A) and genetic evidence indicates that the pathways of prismatic and druse crystal deposition are independent and may even utilize different pathways of oxalate biosynthesis [53]. Thus, the findings presented as a part of this study extends our understanding of the regulation of calcium oxalate crystal formation by supporting a role for *Mt*AAE3 in the regulation of druse crystal accumulation in the different tissues but not prismatic crystal accumulation.

## Conclusion

In this study we provide evidence suggesting that calcium oxalate crystal accumulating plants such as *M*. *truncatula*, utilize the CoA-dependent pathway of oxalate inactivation. Enzyme assays showed *Mt*AAE3 encodes an oxalyl-CoA that is capable of catalyzing the first step in the degradation of oxalate to CO_2_. Expression of this enzyme was also found to be inducible by the substrate, oxalate. This enzyme appears to be essential in the degradation of oxalate whether from an endogenous or exogenous source. The ability to degrade endogenous oxalate was found to be important in the regulation of druse crystal accumulation while the ability to degrade exogenous oxalate was shown to be important in defense against oxalate-secreting phytopathogens. Because of these two functional roles further study of AAE3 and remaining steps of this CoA-dependent pathway of oxalate degradation could lead to the development of new strategies to improve the nutrition quality and production of crops.
